# Circulating Exosomal miRNA Profiles in Non-Small Cell Lung Cancers

**DOI:** 10.3390/cells13181562

**Published:** 2024-09-17

**Authors:** Abeer A. I. Hassanin, Kenneth S. Ramos

**Affiliations:** 1 Center for Genomic and Precision Medicine, Texas A&M Institute of Biosciences and Technology, Texas Medical Center, Houston, TX 77030, USA; ahassanin@tamu.edu; 2 Department of Animal Wealth Development, Faculty of Veterinary Medicine, Suez Canal University, Ismailia 41522, Egypt

**Keywords:** exosomes, miRNAs, NSCLC, diagnostic performance, gene ontology

## Abstract

A growing number of studies have shown that microRNAs (miRNAs) can exert oncogenic or tumor suppressor activities in a variety of cancers, including lung cancer. Given their presence in exosome preparations, microRNA molecules may in fact participate in exosomal intercellular transfers and signaling. In the present study, we examined the profile of 25 circulating exosomal microRNAs in ostensibly healthy controls compared to patients with squamous cell lung cancers (SQCLC) or lung adenocarcinomas (LUAD). Eight miRNAs, namely, miR-21-5p, miR-126-3p, miR-210-3p, miR-221-3p, Let-7b-5p, miR-146a-5p, miR-222-3p, and miR-9-5p, were highly enriched in the cohort and selected for further analyses. All miRNAs were readily detected in non-small cell lung cancer (NSCLC) patients of both sexes at all cancer stages, and their levels in exosomes correlated with the clinicopathological characteristics of tumors. Thus, the presence of these miRNAs in circulating exosomes may contribute to the regulation of oncogenic activity in patients with NSCLC.

## 1. Introduction

Despite extensive research conducted over the past two decades, cancer continues to be a dominant cause of death, ranking as the second highest cause of mortality in the United States [1,2]. Global cancer statistics in 2024 show that lung cancer accounts for the second highest number of cancer-related deaths, with over 340 individuals dying daily from this disease [3]. Therefore, the development of reliable and effective tools to evaluate lung cancer continues to be a priority.

Exosomes are small membrane-bound vesicles that contain proteins, RNAs and DNA fragments implicated in intercellular communication in cancer [4,5]. These vesicles range in diameter from 30 to 150 nanometers and form early multivesicular bodies through sprouting that upon fusion with the plasma membrane create an intracellular vesicle that is subsequently discharged into the extracellular milieu [4,6]. The molecular composition of exosomes reflects the intracellular condition of their source cells, with mounting evidence showing that the intracellular state of cancer-derived exosomes is strikingly the same as that of their mother cells [7,8]. 

MicroRNAs (miRNAs) are short sequences of non-coding RNA (20–24 nucleotides) that play crucial regulatory functions in both unicellular and multicellular eukaryotes [9,10]. miRNAs can recognize and bind to complementary sites in the 3′ untranslated region of the target mRNA, resulting in post-transcriptional gene silencing through degradation of the target or inhibition of translation, depending on the degree of complementarity between the miRNA and the target site [11]. In contrast to mRNA, miRNAs are distinguished by their relative stability, small size, and remarkable regulatory control over gene expression, making them attractive candidate molecules for regulatory control in cancer [12]. 

The present study was conducted to evaluate the exosomal miRNA content of plasma exosomes in ostensibly healthy individuals compared to patients with SQCLC or LUAD. Our initial analysis focused on 25 miRNAs previously shown to correlate with NSCLC [13] and streamlined to eight miRNAs found to be highly abundant in the plasma exosomes of NSCLC patients. We then examined in silico the role of those eight miRNA target genes in regulating the oncogenic activity of NSCLC and the potential utility of exosomal miRNAs as biomarkers of non-small cell lung cancer (NSCLC). 

## 2. Material and Methods

### 2.1. Exosome Isolation and Characterization

Exosomes were isolated by centrifugation from 1 mL of plasma samples. Plasma was centrifuged at 16,000× *g* for 10 min at 4 °C to eliminate remaining cells, debris, apoptotic bodies, and nuclei. The clarified supernatant was transferred to a fresh tube and exosomes processed using the exoEasy Maxi Kit (Qiagen, Hilden, Germany, catalog number: 76064) according to the manufacturer’s instructions. The NanoSight NS300 instrument (NTA) from Nanosight, Morganville, NJ, USA was used for physical characterization and exosome protein markers detected by Western blot analysis.

### 2.2. Plasma Samples and Patients’ Clinical Characteristics

A training cohort of 72 human plasma samples included 24 from ostensibly healthy controls (Cont), 24 from patients diagnosed with SQCLC, and 24 from patients diagnosed with LUAD. Control samples were sourced from BioIVT in Westbury, NY, USA, while NSCLC samples were obtained from Precision for Medicine in Norton, MA, USA. Participants were selected based on gender, age, smoking status, and cancer stage based on the American Joint Committee on Cancer (AJCC) TNM system [14]. Supplementary Materials Table S1 details the demographic and clinicopathological characteristics of subjects in the training cohort. An independent cohort of 36 samples (12 Cont, 12 SQCLC patients, and 12 LUAD patients) sourced from Precision for Medicine (Norton, MA, USA) was used as an external validation cohort. Their clinicopathological characteristics are summarized in Supplementary Materials Table S2. 

### 2.3. Exosomal Mature miRNAs Levels Using Realtime-PCR

Total RNA was extracted using TRIzol reagent (Invitrogen, Carlsbad, CA, USA) according to the manufacturer’s instructions. Exosomes were lysed with TRIzol reagent at room temperature for 5 min, followed by the addition of chloroform for 10 min. After centrifugation at 10,000× *g* for 5 min at 4 °C, the aqueous phase was collected, mixed with isopropanol, and centrifuged again at 12,000× *g* for 30 min at 4 °C. The resulting pellets were washed with 70% ethanol and dissolved in RNase-free water. The Reverse Transcription System (Promega, Madison, WI, USA) was used to synthesize cDNA from 500 ng of each RNA sample. Realtime PCR reaction was performed using the CFX96 Touch Real-Time PCR Detection System (Biorad, Hercules, CA, USA). Two specific primers sets were used for the reference gene (U6) and one universal reverse primer and one specific sense primer for each miRNA molecule (Supplementary Materials Table S3). The A 96-well optical plate was used to incubate the reactions at 95 °C for 10 min, followed by 40 cycles of 95 °C for 15 s and 60 °C for 1 min. Each reaction was performed in triplicate and fold changes were calculated using the 2^∆∆Ct method (where Ct is threshold cycle).

### 2.4. Enrichment Analysis of miRNA Targets Using the CancerMIRNome Database

The gene ontology (GO) functional enrichment analysis and Kyoto Encyclopedia of Genes and Genomes (KEGG) pathway enrichment analysis on plasma exosomal-derived miRNAs target genes were conducted using the CancerMIRNome online database of miRNome profiles of human cancer (http://bioinfo.jialab-ucr.org/CancerMIRNome/, accessed on 28 August 2023). GO functional enrichment analysis was completed using the terms biological process (BP), cellular component (CC), and molecular function (MF). Each miRNA was uploaded individually for exploration using the default settings, and only enriched terms with *p* < 0.05 were considered significant. The top 30 pathways/ontologies were bubble-plotted. 

### 2.5. Functional miRNAs-Target Interactions Network Construction and Visualization

The validated targets, including gene targets associated with NSCLC for each of the eight plasma exosomal miRNAs studied were created and visualized using the miRNet 2.0 web-based platform [15]. The Target Genes network associated with NSCLC and their roles in KEGG pathways was created using the KEGG PATHWAY Database (https://www.genome.jp/kegg/pathway.html, 3 September 2023).

### 2.6. Diagnostic Values of miRNAs for SQCLC and LUAD Using ROC Curve Analysis

The potential diagnostic utility of individual exosomal miRNA levels in SQCLC and LUAD, as well as their respective combinations, was assessed using receptor operating characteristic (ROC) curve analysis. The analyses included area under the curve (AUC), specificity, sensitivity, and criteria calculations. Logistic regression was used to compare the AUC values.

### 2.7. Statistical Analyses

The statistical analyses were conducted using GraphPad Prism, version 9.5.0 (GraphPad Software, San Diego, CA, USA). The ANOVA and Tukey’s HSD test were used to assess variations in plasma exosomal miRNA levels. The levels of miRNAs between subgroups were compared using unpaired *t*-test analysis, and outliers were identified using Grubbs’ test. We removed any data points that were considered significant outliers at *p* < 0.05 from the statistical analysis. For all analyses, a significance level of *p* < 0.05 was used to determine statistical significance, while a significance level of *p* < 0.01 was used to indicate high statistical significance.

## 3. Results

### 3.1. Exosome Characterization

**Figure 1:**
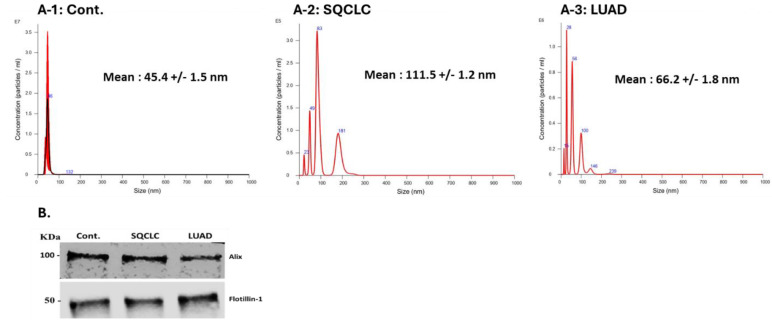
Exosome concentration and diameter in: (**A-1**) Cont. group, (**A-2**) SQCLC patients’ group, (**A-3**) LUAD patients’ group. (**B**) Western blot analysis of exosome protein markers.

**Figure 2:**
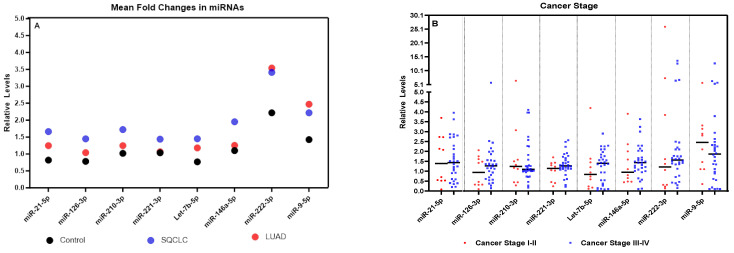

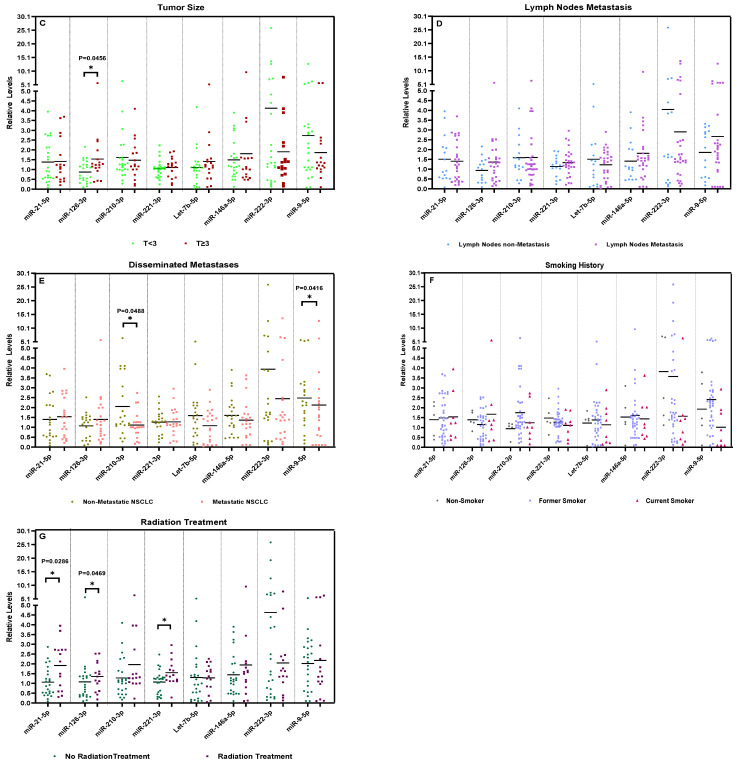
(**A**) Mean fold exosomal miRNA changes in ostensibly healthy controls and patients with SQCLC and LUAD, (**B**–**G**) Correlation between exosomal miRNAs and clinicopathologic characteristics. (**B**) Levels of the eight microRNAs did not differ significantly with respect to cancer stage. (**C**) NSCLC patients with T ≥ 3 showed significantly high levels of exosomal miR-126-3p (*p* = 0.0456). (**D**) Lymph node metastasis did not correlate with the levels of the eight miRNAs. (**E**) Non-metastatic NSCLC tumors exhibited significantly high levels of exosomal miR-210-3p (*p* = 0.0488) and miR-9-5p (*p* = 0.0416). (**F**) Levels of the eight microRNAs were not significantly impacted by smoking status. (**G**) The levels of miR-21-5p (*p* = 0.0286), miR-126-3p (*p* = 0.0469), and miR-221-3p (*p* = 0.0462) were elevated in those patients who underwent radiation therapy. * *p* < 0.05.

**Figure 3:**
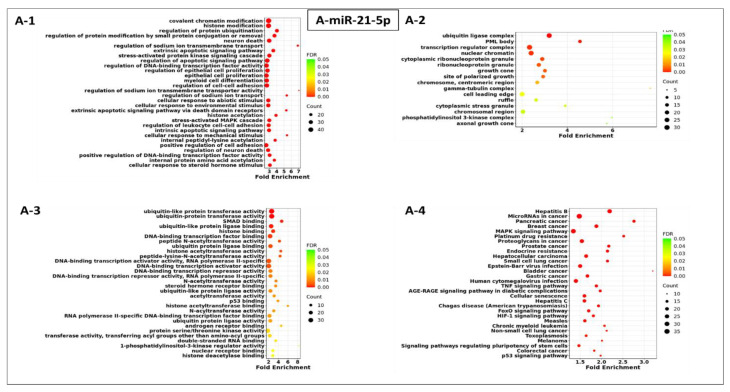

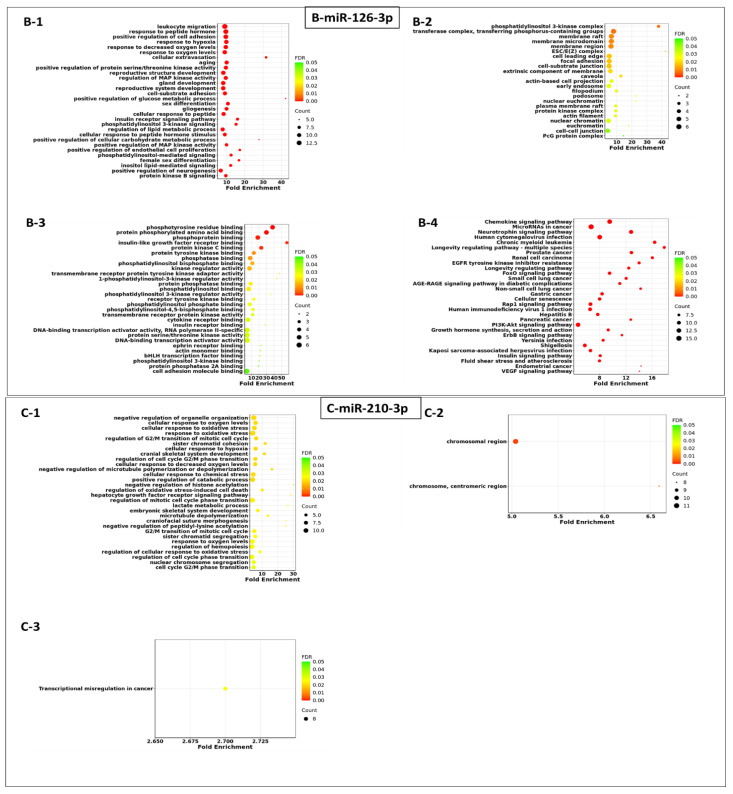

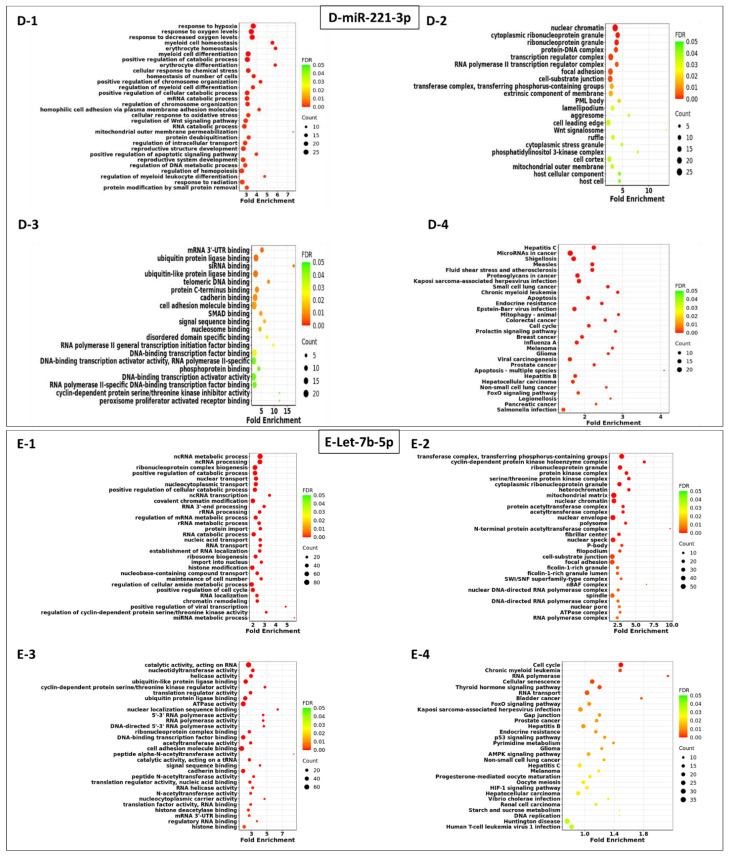

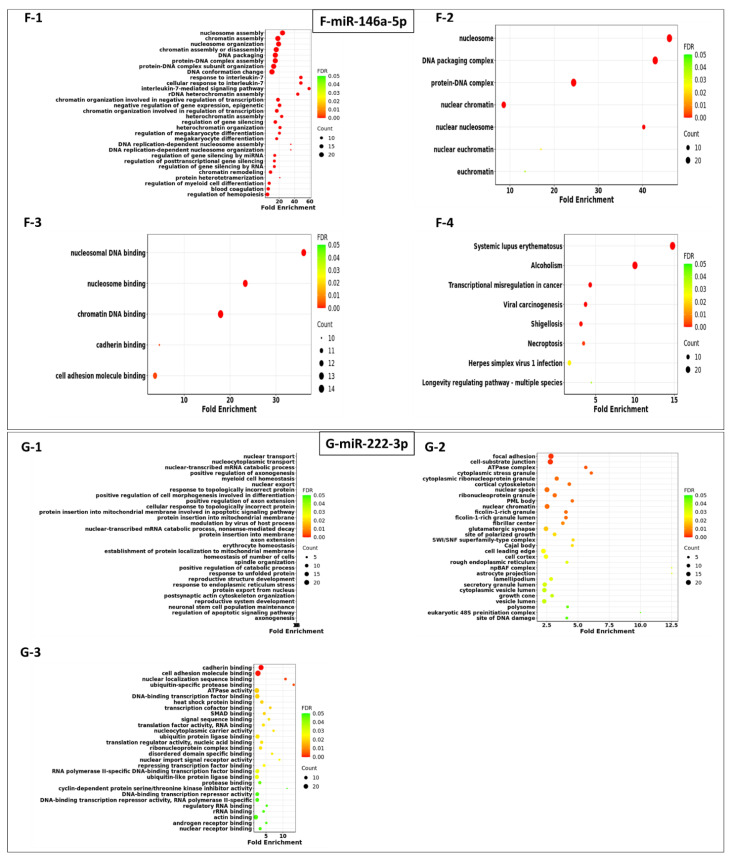

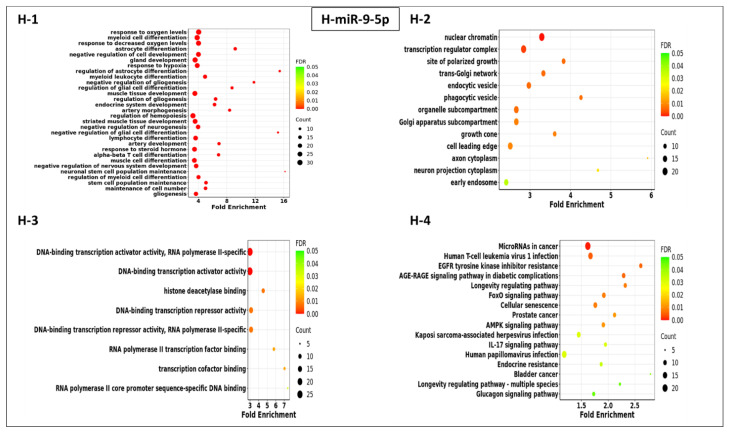
Gene ontology and KEGG pathway enrichment analyses; (**A**): Gene ontology and KEGG pathway enrichment analysis of miR-21-5p target genes: The top 30 gene ontology enrichment results (**A-1**) BP, biological process, (**A-2**) CC, cellular component, (**A-3**) MF, molecular function, (**A-4**) KEGG enrichment pathways. (**B**): Gene ontology and KEGG pathway enrichment analysis of miR-126-3p target genes: The top 30 gene ontology enrichment results (**B-1**) BP, biological process, (**B-2**) CC, cellular component, (**B-3**) MF, molecular function, (**B-4**) KEGG enrichment pathways. (**C**): Gene ontology and KEGG pathway enrichment analysis of miR-210-3p target genes: The top 30 gene ontology enrichment results (**C-1**) BP, biological process, (**C-2**) CC, cellular component, (**C-3**) KEGG enrichment pathways. (**D**): Gene ontology and KEGG pathway enrichment analysis of miR-221-3p target genes: The top 30 gene ontology enrichment results (**D-1**) BP, biological process, (**D-2**) CC, cellular component, (**D-3**) MF, molecular function, (**D-4**) KEGG enrichment pathways. (**E**): Gene ontology and KEGG pathway enrichment analysis of Let-7b-5p target genes: The top 30 gene ontology enrichment results (**E-1**) BP, biological process, (**E-2**) CC, cellular component, (**E-3**) MF, molecular function, (**E-4**) KEGG enrichment pathways. (**F**): Gene ontology and KEGG pathway enrichment analysis of miR-146a-5p target genes: The top 30 gene ontology enrichment results (**F-1**) BP, biological process, (**F-2**) CC, cellular component, (**F-3**) MF, molecular function, (**F-4**) KEGG enrichment pathways. (**G**): Gene ontology and KEGG pathway enrichment analysis of miR-222-3p target genes: The top 30 gene ontology enrichment results (**G-1**) BP, biological process, (**G-2**) CC, cellular component, (**G-3**) MF, molecular function. (**H**): Gene ontology and KEGG pathway enrichment analysis of miR-9-5p target genes: The top 30 gene ontology enrichment results (**H-1**) BP, biological process, (**H-2**) CC, cellular component, (**H-3**) MF, molecular function, (**H-4**) KEGG enrichment pathways. False discovery rate (FDR) and the number of enriched genes is represented by the bubble color and size, respectively. Target genes that have been mapped to a certain pathway as a proportion of all genes within that pathway are referred to as fold enrichment.

Exosome concentrations and diameters for each group are shown in Figure 1A-1–A-3. Western blot results of the exosome protein markers Alix and Flotillin-1 confirmed the exosomal identity of all preparations used in our studies (Figure 1B).

### 3.2. Exosomal miRNA Profiles

Exosomes from female and male patients with SQCLC and LUAD showed differences in eight mature exosomal miRNAs compared to controls (Supplementary Materials Table S4 and Figure 2A). Exosomes from females with SQCLC showed significantly higher levels of miR-21-5p (*p* = 0.0138), miR-126-3p (*p* = 0.0196), miR-221-3p (*p* = 0.0180), and miR-146a-5p (*p* = 0.0461) (Supplementary Materials Figures S1A-1, S1A-2, S1A-4 and S1A-6, respectively), while exosomes from SQCLC males showed significantly higher levels of miR-210-3p (*p* = 0.0320), Let-7b-5p (*p* = 0.0180), and miR-9-5p (*p* = 0.0241) (Supplementary Materials Figures S1B-3, S1B-5 and S1B-8, respectively). SQCLC exosomes for both genders contained significantly higher levels of miR-21-5p (*p* = 0.0050), miR-126-3p (*p* = 0.0050), miR-210-3p (*p* = 0.0129), miR-221-3p (*p* = 0.0017), let-7b-5p (*p* = 0.0050), miR-146a-5p (*p* = 0.0129), miR-222-3p (*p* = 0.0310), and miR-9-5p (*p* = 0.0129) compared to controls (Supplementary Materials Figure S1C1–C8, respectively). Exosomes from LUAD patients of both genders showed significantly higher levels of miR-221-3p (*p* = 0.0129) and miR-222-3p (*p* = 0.0310) compared to controls (Supplementary Materials Figures S1C-4 and S1C-7, respectively). Thus, variation in exosomal miRNA profiles between SQCLC and LUAD patients may reveal functional differences between NSCLC histological subtypes in the regulation of genes that drive specific molecular phenotypes. 

We next examined the relationship between exosomal miRNA levels and tumor stage and size, lymph node metastases, tumor metastases, smoking habits, and radiation treatment (Figure 2B–G). Significantly elevated levels of miR-126-3p were found in NSCLC patients with tumor sizes T ≥ 3 (*p* = 0.0456) (Figure 2C). Additionally, miR-210-3p (*p* = 0.0488), and miR-9-5p (*p* = 0.0416) were highly expressed in non-metastatic NSCLC tumors (Figure 2E). Significant increases in miR-21-5p (*p* = 0.0286), miR-126-3p (*p* = 0.0469), and miR-221-3p (*p* = 0.0462) levels were seen in patients who underwent radiation treatment (Figure 2G). Thus, the levels of exosomal miR-126-3p, miR-210-3p, and miR-9-5p may effectively distinguish clinicopathological characteristics among NSCLC patients. 

### 3.3. Enrichment of miRNA Target Genes and Functional Interaction Networks

**Figure 4:**
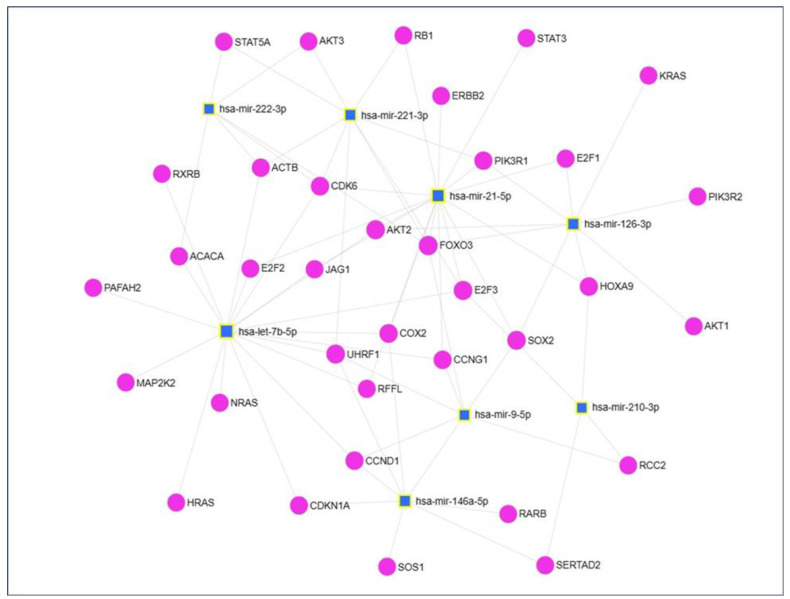
miRNA-target genes network diagram of plasma exosomal-derived miRNAs molecules; Eleven of the verified targets for miR-21-5p (CDK6, ERBB2, E2F2, STAT3, PIK3R1, AKT2, RB1, EGFR, E2F1, E2F3, FOXO3) were associated with NSCLC, six of the miR-126-3p targets (KRAS, PIK3R1, AKT2, FOXO3, PIK3R2, AKT1) were associated with NSCLC, for miR-210-3p, E2F3 was shown to be associated with NSCLC, seven verified miR-221-3p targets (PIK3R1, STAT5A, RB1, AKT3, CDK6, E2F3, and FOXO3) were linked with NSCLC, nine Let-7b-5p targets (HRAS, E2F2, RXRB, MAPK1, CCND1, AKT2, NRAS, CDKN1A, MAP2K2) were linked to NSCLC, four miR-146a-5p targets (SOS1, RARB, CCND1, and EGFR) were associated to NSCLC, NSCLC was linked to miR-222-3p gene targets (TP53, STAT5A, AKT3, CDK6, and FOXO3), miR-9-5p validated targets FOXO3 and CCND1 were associated to NSCLC. Blue box nodes represent the miRNAs and pink dot nodes denote their target genes.

Figure 3A–H display the GO terms and KEGG pathways for putative miRNA target genes. Regarding biological processes (BP), the terms enriched included covalent chromatin modification, leukocyte migration, negative regulation of organelle organization, response to hypoxia, ncRNA metabolic process, nucleosome assembly, nuclear transport, and myeloid cell differentiation (Figure 3A-1,B-1,C-1,D-1,E-1,F-1,G-1,H-1). For the cellular component (CC) analyses, target genes enriched included the ubiquitin ligase complex, phosphatidylinositol 3-kinase complex, chromosomal region, nuclear chromatin, the transferase complex, transferring phosphorus-containing groups, the nucleosome, focal adhesion, and nuclear chromatin (Figure 3A-2,B-2,C-2,D-2,E-2,F-2,G-2,H-2). Lastly, in terms of molecular functions (MF), the terms enriched included ubiquitin-like protein transferase activity, phosphotyrosine residue binding, mRNA 3′-UTR binding, catalytic activity acting on RNA, nucleosomal DNA binding, cadherin binding, and DNA-binding transcription activator activity (Figure 3A-3,B-3,C-3,D-3,E-3,F-3,G-3,H-3). KEGG pathway analysis showed that the eight highly expressed miRNAs were primarily involved in the Hepatitis B, chemokine signaling pathway, microRNA in cancer pathway, transcriptional misregulation in cancer, Hepatitis C, cell cycle, systemic lupus erythematosus pathway, and alcoholism pathway (Figure 3A-4,B-4,D-4,E-4,F-4,H-4).

**Figure 5:**
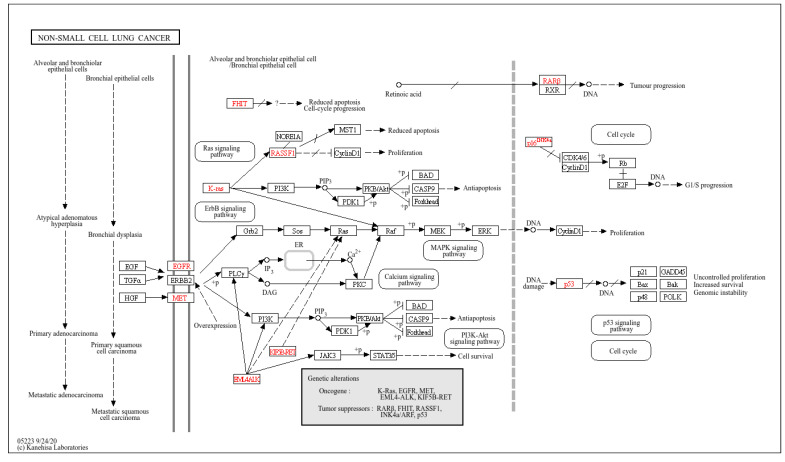
KEGG pathway of the putative biological roles of miRNAs-targets correlated with NSCLC. The arrows display all KEGG relationships such as macromolecular complex activation, combined activation, inhibition, and activation. Indirect and missing interactions are also illustrated. Red color represents both oncogenes and tumor suppressor genes.

As cancers and NSCLC were among the top 30 KEGG pathways targeted by the eight plasma exosomal miRNAs (Figure 3A-4, B-4, D-4, E-4, F-4, H-4), predicted miRNA target genes (including those associated with NSCLC) were retrieved from the miRNet2.0 database. These included CDK6, ERBB2, E2F2, STAT3, PIK3R1, AKT2, RB1, EGFR, E2F1, E2F3, FOXO3, KRAS, PIK3R2, AKT1, STAT5A, AKT3, HRAS, RXRB, MAPK1, CCND1, NRAS, CDKN1A, MAP2K2, SOS1, RARB, and TP53 (Figure 4). The putative biological role of these target genes in NSCLC was revealed by KEGG pathway analysis (Figure 5), in keeping with previous work by our group linking regulatory control of LINE-1, RB1, EGFR, E2F1, and TP53 [16,17,18]. 

## 4. Exosomal miRNAs as Cancer Biomarkers

**Figure 6:**
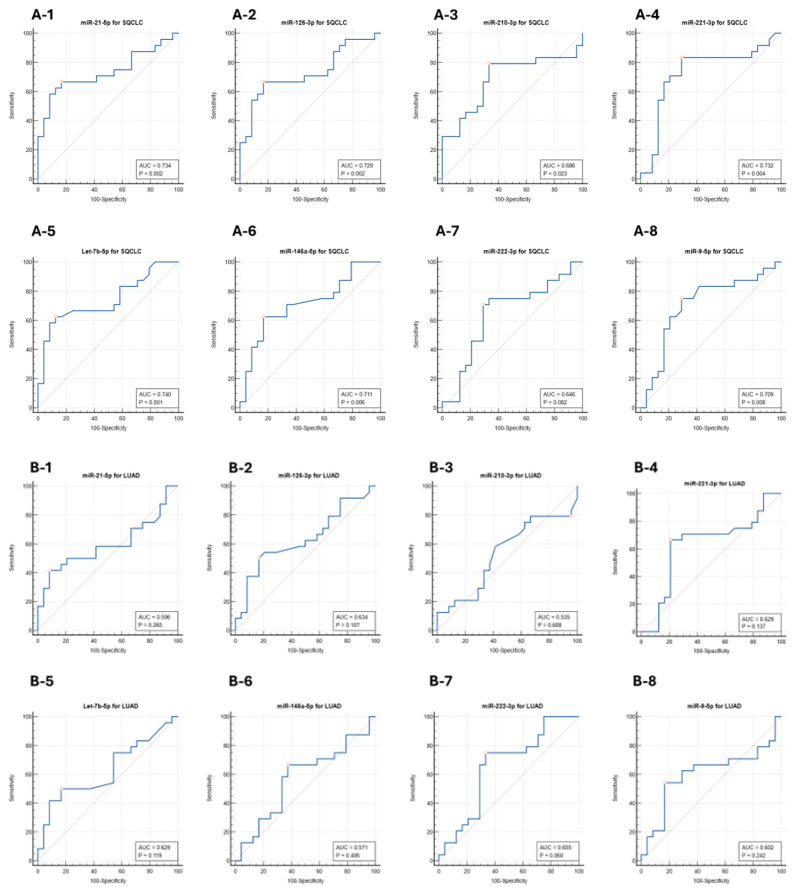

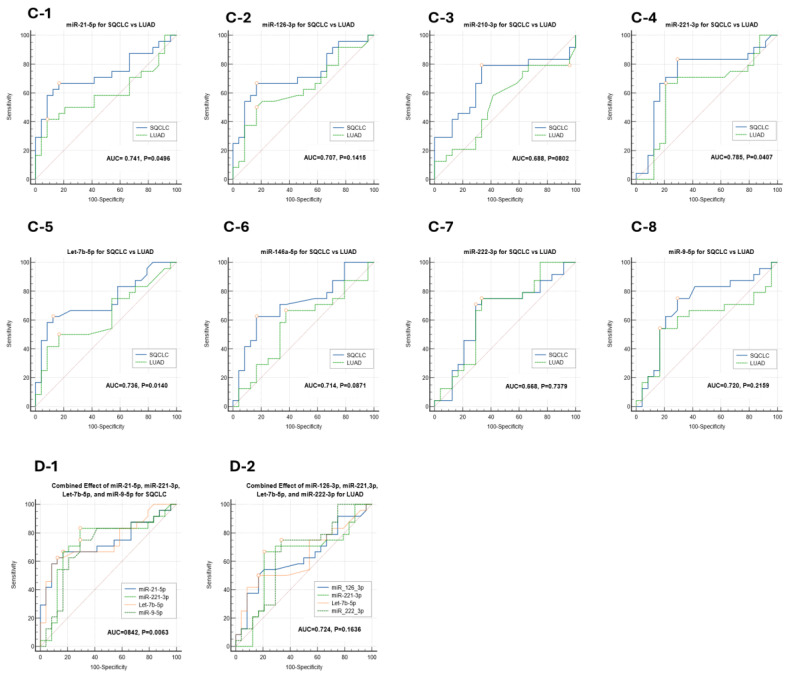
(**A**); ROC curves of plasma exosomal miRNAs in SQCLC patients in the training cohort; (**A-1**) miR-21-5p, (**A-2**) miR-126-3p, (**A-3**) miR-210-3p, (**A-4**) miR-221-3p, (**A-5**) Let-7b-5p, (**A-6**) miR-146a-5p, (**A-7**) miR-222-3p, and (**A-8**) miR-9-5p. (**B**); ROC curves of plasma exosomal miRNAs in LUAD patients in the training cohort; (**B-1**) miR-21-5p, (**B-2**) miR-126-3p, (**B-3**) miR-210-3p, (**B-4**) miR-221-3p, (**B-5**) Let-7b-5p, (**B-6**) miR-146a-5p, (**B-7**) miR-222-3p, and (**B-8**) miR-9-5p. (**C**); ROC curves of plasma exosomal miRNAs to distinguish patients with SQCLC from patients with LUAD in the training cohort; (**C-1**) miR-21-5p, (**C-2**) miR-126-3p, (**C-3**) miR-210-3p, (**C-4**) miR-221-3p, (**C-5**) Let-7b-5p, (**C-6**) miR-146a-5p, (**C-7**) miR-222-3p, and (**C-8**) miR-9-5p. (**D**); ROC curve analysis of four exosomal miRNA panels were combined to distinguish: (**A**) SQCLC patients from healthy control group, (**B**) LUAD patients from healthy control group.

AUC values for SQCLC patients compared to controls ranged from 0.646 to 0.740. Selected miRNAs showed sensitivities ranging from 62.5 to 79.2% and specificities ranging from 66.7 to 87.5% at the *p* < 0.05 level (Table 1 and Figure 6A-1–A-8). ROC curve analyses for LUAD patients compared to controls showed non-significant AUC values ranging from 0.535 to 0.655 with sensitivities ranging from 50.0 to 79.2% and specificities ranging from 4.2 to 83.3% (Table 1 and Figure 6B-1–B-8). Univariate logistic regression was used to determine whether the eight exosomal miRNAs identified could distinguish SQCLC from LUAD using controls as a reference. The AUC values for all eight miRNAs ranged from 0.668 to 0.785 (Table 1 and Figure 6C-1–C-8), with a significant ratio for miR-21-5p (AUC = 0.741; *p* = 0.0496) (Figure 6C-1), miR-221-5p (AUC = 0.785; *p* = 0.0407) (Figure 6C-4) and Let-7b-5p (AUC = 0.736; *p* = 0.0140) (Figure 6C-5). The combined diagnostic performance of miR-21-5p, miR-221-5p, Let-7b-5p, and miR-9-5p was stronger than that of any individual miRNA, with significant differences seen for SQCLC patients compared to controls (AUC = 0.842; *p* = 0.0063) (Figure 6D-1). The combined efficacy of miR-126-3p, miR-221-5p, Let-7b-5p, and miR-222-3p in differentiating LUAD patients from controls was also superior to that of any single miRNA (Figure 6D-2). Based on these findings, we conclude that modest improvements in biomarker accuracy may be realized when the signals for miR-21-5p, miR-221-5p, Let-7b-5p, and miR-9-5p exosomal miRNAs are combined. 

## 5. External Validation of Training Set Findings

We applied our model to an independent validation cohort to confirm the robustness and generalizability of findings (Supplementary Materials Figure S2A–D). AUC values, sensitivity, specificity, and all cut-off values were calculated using ROC analysis and summarized in Table 2. The eight exosomal miRNAs exhibited AUC values ranging from 0.646 to 0.788 in differentiating SQCLC patients from controls (Table 2 and Supplementary Materials Figure S2A-1–A-8), For AUC values ranged from 0.524 to 0.681 differentiating LUAD patients from controls (Table 2 and Supplementary Materials Figure S2B-1–B-8). AUC values ranged from 0.653 to 0.792 differentiating between SQCLC and LUAD patients (Table 2, Supplementary Materials Figure S2C-1–C-8). The combined diagnostic efficacy of miR-21-5p, miR-221-5p, Let-7b-5p, and miR-9-5p distinguishing SQCLC patients from controls was 0.842 and found to be superior to those when all miRNAs were combined (Supplementary Materials Figure S2D-1). In addition, the combined diagnostic performance of miR-126-3p, miR-221-5p, Let-7b-5p, and miR-222-3p at 0.764 was superior in distinguishing LUAD patients from controls (Supplementary Materials Figure S2D-2). These findings collectively validate the effectiveness and reliability of the model in distinguishing between controls and the two predominant histological subtypes of NSCLC.

## 6. Discussion

The study of exosomal miRNAs and their role in the regulation of oncogenic signaling may shed light into critical pathways of lung oncogenesis. miR-210 has been associated with the loss of mitochondrial membrane potential and the acquisition of abnormal mitochondrial phenotypes that compromise HIF-1 activity, cellular metabolism, and survival [19]. Let-7b miRNA negatively regulates oncogenes like RAS, MYC, and HMGA2, as well as cell-cycle progression genes like CDC25A, CDK6, and cyclin D2 [20,21,22,23,24]. Exosomal Let-7b has been proposed as a diagnostic biomarker for NSCLC [25], with downregulation linked to poor prognosis [26]. miR-222 downregulates the cyclin-dependent kinase (CDK) inhibitors p27Kip1 and p57 and upregulates the epithelial-to-mesenchymal transition gene ZEB2, with changes being developed as a liquid biopsy for early-stage NSCLC [27,28], miR-221 promotes apoptosis of NSCLC cells through negative regulation of lncRNA HOTAIR [29], and miR-9 promotes lung cancer cell invasion and adhesion [30]. EV miR-21 activates Toll-like receptors in immune cells, releasing inflammatory cytokines such as TNF-α and IL-6 and eliciting a pro-metastatic inflammatory response [31]. Lastly, NSCLC cells express miR-146a at higher levels than normal lung cells [32], a pattern associated with shifts in cell proliferation and metastasis and induction of apoptosis via EGFR signaling [33]. Overall, the observed profiles suggest that the eight exosomal miRNAs identified may play crucial roles in the pathogenesis and progression of NSCLC. This interpretation is consistent with the strong correlation of these miRNAs with the clinicopathological characteristics of NSCLC patients. 

SQCLC patients showed significantly higher levels of all eight miRNAs compared to controls, and LUAD patients showed significantly higher levels of mR-221-3p and miR-222-3p. Ref. [34] reported similar findings, stating that exosomes produced by the LUAD cell line (A549) can transport miR-222-3p to target cells, promoting cell proliferation, migration, and invasion by targeting suppressor of cytokine signaling 3 (SOCS3). Further, exosomal levels of miR-126-3p, miR-210-3p, and miR-9-5p were also associated with clinicopathological characteristics. Exosomal miR-9 activates the JAK/STAT pathway, which, in turn, increases angiogenesis, a critical event in the development and spread of tumors [35]. NSCLC cells release exosomal miR-126, which can induce angiogenesis and accelerate the NSCLC progression [36]. Furthermore, miR-210 enclosed within exosomes derived from tumor cells promotes the process of angiogenesis [37]. Our results also showed a strong correlation between exosomal miR-21-5p, miR-126-3p, and miR-221-3p miRNAs and radiotherapy treatment. Similar findings were reported by others, showing that activation of the PI3K/AKT/mTOR signaling pathway by miR-21 may reduce PDCD4 expression, which, in turn, may alter radiation sensitivity in NSCLC cells [38]. miR-126 plays a role in controlling the response of NSCLC cells to cancer treatment and can promote radiation-induced NSCLC cell apoptosis by activating the PI3K-Akt pathway [39]. ROC curves for exosomal miRNAs in SQCLC and LUAD patients distinguished SQCLC and LUAD cases from controls. The combination of miR-21-5p, miR-221-3p, let-7b-5p, and miR-9-5p for SQCLC and miR-126-3p, miR-221-3p, let-7b-5p, and miR-222-3p for LUAD yielded superior diagnostic performance compared to single miRNAs or alternative miRNA combinations. Thus, an expanded panel of miRNAs is likely best suited for use in evaluating NSCLC patients, as suggested by [40,41].

## 7. Conclusions

In conclusion, our findings shed light into the putative roles of exosomal miRNAs in NSCLC, and their combined utility in distinguishing primary NSCLC histological subtypes. Furthermore, these findings demonstrate that miR-126-3p, miR-210-3p, and miR-9-5p have a significant role in differentiating the clinicopathological characteristics of NSCLC. Exosomal miR-21-5p, miR-126-3p, and miR-221-3p expression profiles may serve as reliable predictors of response to radiation treatment. Exploring the role of exosomal miRNAs in NSCLC development and progression may open new opportunities for evaluation of the molecular basis of NSCLC.

While our study provides valuable insights into the potential diagnostic utility of exosomal miRNAs in the evaluation of NSCLC patients, several characteristics may limit the generalizability of findings. The study was limited to a relatively small number of samples and no information was available on the geographic origin of patients or their chemotherapy status. Additionally, we restricted our analysis to miRNAs previously linked to NSCLC and this may have excluded important signals. Thus, future studies are needed to explore a broader range of miRNAs using a cross-sectional design for longitudinal evaluation of the prognostic utility of the identified miRNAs. It would also be of interest to evaluate the functional impact of miRNAs on NSCLC pathology relative to the status of driver oncogene mutations to reveal additional regulatory pathways, track changes in miRNA profiles over time, and gain deeper insights into the prognostic value of the miRNAs.

## Figures and Tables

**Table 1:** Diagnostic performance of the eight exosomal miRNAs in the training cohort.

miRNA	SQCLC				LUAD				SQCLC vs. LUAD
	AUC (95% CI)	Se	Sp	Criterion	AUC (95% CI)	Se	Sp	Criterion	AUC (95% CI)
miR-21-5p	0.734 (0.587 to 0.851)	66.7	83.3	>1.09	0.596 (0.445 to 0.735)	41.7	91.7	>1.23	0.741 (0.595 to 0.857)
miR-126-3p	0.729 (0.582 to 0.847)	66.7	83.3	>1.08	0.643 (0.482 to 0.768	50	83.3	>1.08	0.707 (0.558 to 0.829)
miR-210-3p	0.686 (0.536 to 0.812)	79.2	66.7	>1.03	0.535 (0.385 to 0.680)	79.2	4.2	>0.46	0.688 (0.537 to 0.813)
miR-221-3p	0.732 (0.584 to 0.849)	83.3	70.8	>1.01	0.629 (0.478 to 0.764)	66.7	79.2	>1.1	0.785 (0.642 to 0.890)
Let-7b-5p	0.740 (0.593 to 0.855)	62.5	87.5	>1.26	0.629 (0.478 to 0.7640	50	83.3	>1.2	0.736 (0.589 to 0.853)
miR-146a-5p	0.711 (0.562 to 0.833)	62.5	83.3	>1.4	0.571 (0.420 to 0.713)	66.7	62.5	>1	0.714 (0.565 to 0.835)
miR-222-3p	0.646 (0.495 to 0.778)	70.8	70.8	>1.25	0.655 (0.503 to 0.786)	75	66.7	>1.03	0.668 (0.518 to 0.797)
miR-9-5p	0.709 (0.560 to 0.831)	75	70.8	>1.25	0.602 (0.451 to 0.741)	54.2	83.3	>1.86	0.720 (0.572 to 0.840)

**Table 2:** Diagnostic performance of the eight exosomal miRNAs in the validation cohort.

miRNA	SQCLC				LUAD				SQCLC vs. LUAD
	AUC (95% CI)	Se	Sp	Criterion	AUC (95% CI)	Se	Sp	Criterion	AUC (95% CI)
miR-21-5p	0.788 (0.575 to 0.927)	75	91.7	>1	0.524 (0.313 to 0.730)	41.7	91.7	≤0.62	0.792 (0.578 to 0.929)
miR-126-3p	0.722 (0.503 to 0.884)	83.3	66.7	>1	0.635 (0.416 to 0.820)	91.7	41.7	>1.07	0.771 (0.555 to 0.916)
miR-210-3p	0.722 (0.503 to 0.884)	83.3	73	>1.12	0.601 (0.383 to 0.793)	66.7	58.3	>1	0.729 (0.511 to 0.888)
miR-221-3p	0.747 (0.529 to 0.900)	75	75	>1.16	0.681 (0.461 to 0.854)	75	66.7	>1.46	0.715 (0.496 to 0.879)
Let-7b-5p	0.764 (0.548 to 0.911)	75	83.3	>1	0.639 (0.419 to 0.823)	83.3	50	>1.03	0.764 (0.548 to 0.911)
miR-146a-5p	0.722 (0.503 to 0.884)	83.3	66.7	>1	0.549 (0.334 to 0.750)	33.3	41.7	>0.84	0.736 (0.518 to 0.893)
miR-222-3p	0.646 (0.426 to 0.828)	75	58.3	>1	0.604 (0.386 to 0.796)	58.3	75	>1	0.653 (0.433 to 0.833)
miR-9-5p	0.743 (0.525 to 0.898)	66.7	91.7	>1.39	0.639 (0.419 to 0.823)	66.7	75	>1.39	0.743 (0.525 to 0.898)

## Data Availability

The data presented in this study are available on request from the corresponding author.

## Supplementary Materials

The following supporting information can be downloaded at: https://www.mdpi.com/article/10.3390/cells13181562/s1.
