# Supplementary material for: Circulating Exosomal miRNA Profiles in Non-Small Cell Lung Cancers
Source: Cells. 2024 Sep 17;13(18):1562. doi: 10.3390/cells13181562 (PMC11430728; doi:10.3390/cells13181562)
Supplement: Supplementary file 1 [file cells-13-01562-s001.zip › cells-3196239-supplementary.pdf]

Supplementary Table S1: Demographic and Clinical Characteristics of Individuals in the CT, SQCLC, and LUAD Groups in the Training Cohort.

Clinical Profile		CT Group (N=24)	NSCLC Groups	
			SQCLC Group (N=24)	LUAD Group (N=24)
Gender (N)	Female	12	12	12
	Male	12	12	12
Age (mean ±SE)		70 ±1.68	72.46±1.94	71.04±1.61
Smoking History (N)	Non-Smoker	N/A	2	3
	Former Smoker	N/A	17	17
	Current Smoker	N/A	5	3
	N/A	N/A	-	1
TNM Staging of NSCLC				
Cancer Stage (N)	Early Stage (Stage I-II)	-	6	4
	Late Stage (Stage III-IV)	-	14	16
	N/A	-	4	4
Tumor Size (N)	Small Sized Tumors (T<3)	-	12	11
	Large Sized Tumors (T≥3)	-	10	7
	N/A	-	2	6
Regional Lymph Nodes Involved (N)	No regional node metastasis	-	9	6
	Regional nodes metastasis	-	14	14
	N/A	-	1	4
Disseminated Metastases (N)	Non-Metastatic	-	14	7
	Metastatic	-	8	13
	N/A	-	2	4
Radiation Treatment (N)				
	NO	-	12	12
	YES	-	9	6
	N/A	-	3	6

Supplementary Table S2: Demographic and Clinical Characteristics of Individuals in the CT, SQCLC, and LUAD Groups in the Validation Cohort.

**Clinical Profile**		**CT (N=12)**	**NSCLC**	
			**SQCLC (N=12)**	**LUAD (N=12)**
Gender (N)	Female	6	6	6
	Male	6	6	6
Age (mean ±SE)		70.63±0.6	73.33±1.67	73.5±1.72
Smoking History (N)	Non-Smoker	N/A	-	1
	Former Smoker	N/A	10	10
	Current Smoker	N/A	1	1
	N/A	N/A	1	-
TNM Staging of NSCLC				
Cancer Stage (N)	Early Stage (Stage I-II)	-	5	1
	Late Stage (Stage III-IV)	-	4	11
	N/A	-	3	-
Tumor Size (N)	Small Sized Tumors (T<3)	-	6	9
	Large Sized Tumors (T≥3)	-	3	2
	N/A	-	3	1
Regional Lymph Nodes Involved (N)	No regional node metastasis	-	4	1
	Regional nodes metastasis	-	5	10
	N/A	-	3	1
Disseminated Metastases (N)	Non-Metastatic	-	7	5
	Metastatic	-	2	6
	N/A	-	3	1
	NO	-	4	1
	YES	-	6	5
	N/A	-	2	6

Supplementary Table S3: miRNAs Primers Sequences

miRNA	Forward Primer (5′-3′)	Reverse Primer (5′-3′)
U6	CTCGCTTCGGCAGCACA	AACGCTTCACGAATTTGCGT
miR-21-5p	ACACTCCAGCTGGGTAGCTTATCA GACTGA	CTCAACTGGTGTCGTGGAGTCGGCAATTCAGT TGAGTCAACATC
miR-126-3p	ACACTCCAGCTGGGTCGTACCGTG AGTAA	CTCAACTGGTGTCGTGGAGTCGGCAATTCAGT TGAGGCATTATT
miR-210-3p	ACACTCCAGCTGGGCTGTGCGTGT GACAGC	CTCAACTGGTGTCGTGGAGTCGGCAATTCAGT TGAGTCAGCCGC
miR-221-3p	ACACTCCAGCTGGGAGCTACATTG TCTGCT	CTCAACTGGTGTCGTGGAGTCGGCAATTCAGT TGAGGAAACCCA
Let-7b-5p	ACACTCCAGCTGGGTGAGGTAGTA GGTTGT	CTCAACTGGTGTCGTGGAGTCGGCAATTCAGT TGAGAACCACAC
miR-146a-5p	ACACTCCAGCTGGGTGAGAACTGA ATTCCA	CTCAACTGGTGTCGTGGAGTCGGCAATTCAGT TGAGAACCCATG
miR-222-3p	ACACTCCAGCTGGGAGCTACATCT GGCTACTG	CTCAACTGGTGTCGTGGAGTCGGCAATTCAGT TGAGGAGACCCA
miR-9-5p	ACACTCCAGCTGGGTCTTTGGTTAT CTAGCT	CTCAACTGGTGTCGTGGAGTCGGCAATTCAGT TGAGTCATACAG

Supplementary Table S4: miRNA Levels in Plasma Exosomes of Ostensibly Healthy Control Subjects and SQCLC, LUAD Patients.

	**Controls (Mean ±SE)**	**SQCLC (Mean ±SE)**	**LUAD (Mean ±SE)**
**miR-21-5p**	0.82±0.11	1.66±0.22	1.25±0.20
**miR-126-3p**	0.78±0.10	1.45±0.23	1.04±0.13
**miR-210-3p**	1.02±0.10	1.72±0.28	1.25±0.21
**miR-221-3p**	1.04±0.15	1.44±0.14	1.07±0.09
**Let-7b-5p**	0.77±0.12	1.45±0.20	1.18±0.21
**miR-146a-5p**	1.10±0.18	1.95±0.39	1.26±0.19
**miR-222-3p**	2.22±0.73	3.41±1.08	3.54±1.01
**miR-9-5p**	1.43±0.28	2.22±0.29	2.47±0.57

Supplementary Figure Legends

Supplementary Figure S1: Relative fold change in expression levels of eight exosomal miRNAs in ostensibly healthy controls (cont.), squamous cell lung cancer patients (SQCLC), and lung adenocarcinoma patients (LUAD); A1-A8 : Relative levels of miR-21-5p (A-1), mir-126-3p (A-2), miR-221-3p (A-4), and miR-146a-5p (A-6) in female SQCLC patients were significantly higher than in controls. B1-B8: Relative levels of miR-210-3p (B-3), Let-7b-5p (4B-5), and mir-9-5p (4B-8) in male SQCLC patients were significantly higher than in controls. C1-C8: Relative levels of miR-21-5p (C-1), miR-126-3p (C-2), miR-210-3p (C-3), miR-221-3p (C-4), let-7b-5p (C-5), miR-146a-5p (C-6), miR-222-3p (C-7), and miR-9-5p (C-8) in both males and female SQCLC patients were significantly higher levels than controls. LUAD patients had significantly higher levels of miR-221-3p (C-4) and miR-222-3p (C-7) than controls.

Supplementary Figure S2: A: ROC Curves of Plasma Exosomal miRNAs in a Validation Cohort of SQCLC Patients. (A-1) miR-21-5p, (A-2) miR-126-3p, (A-3) miR-210-3p, (A-4) miR-221-3p, (A-5) Let-7b-5p, (A-6) miR-146a-5p, (A-7) miR-222-3p, and (A-8) miR-9-5p. **B:** ROC Curves of Plasma Exosomal miRNAs in a Validation Cohort of LUAD Patients. (B-1) miR-21-5p, (B-2) miR-126-3p, (B-3) miR-210-3p, (B-4) miR-221-3p, (B-5) Let-7b-5p, (B-6) miR-146a-5p, (B-7) miR-222-3p, and (B-8) miR-9-5p. **C:** ROC Curves of Plasma Exosomal miRNAs in a Validation Cohort to Distinguish SQCLC from LUAD. (C-1) miR-21-5p, (C-2) miR-126-3p, (C-3) miR-210-3p, (C-4) miR-221-3p, (C-5) Let-7b-5p, (C-6) miR-146a-5p, (C-7) miR-222-3p, and (C-8) miR-9-5p. **D:** Combined ROC Curve Analysis of Four Selected Exosomal miRNA to Differentiate NSCLC Subtypes from Controls. A) SQCLC; B) LUAD.

Supplementary Figure S1.

A

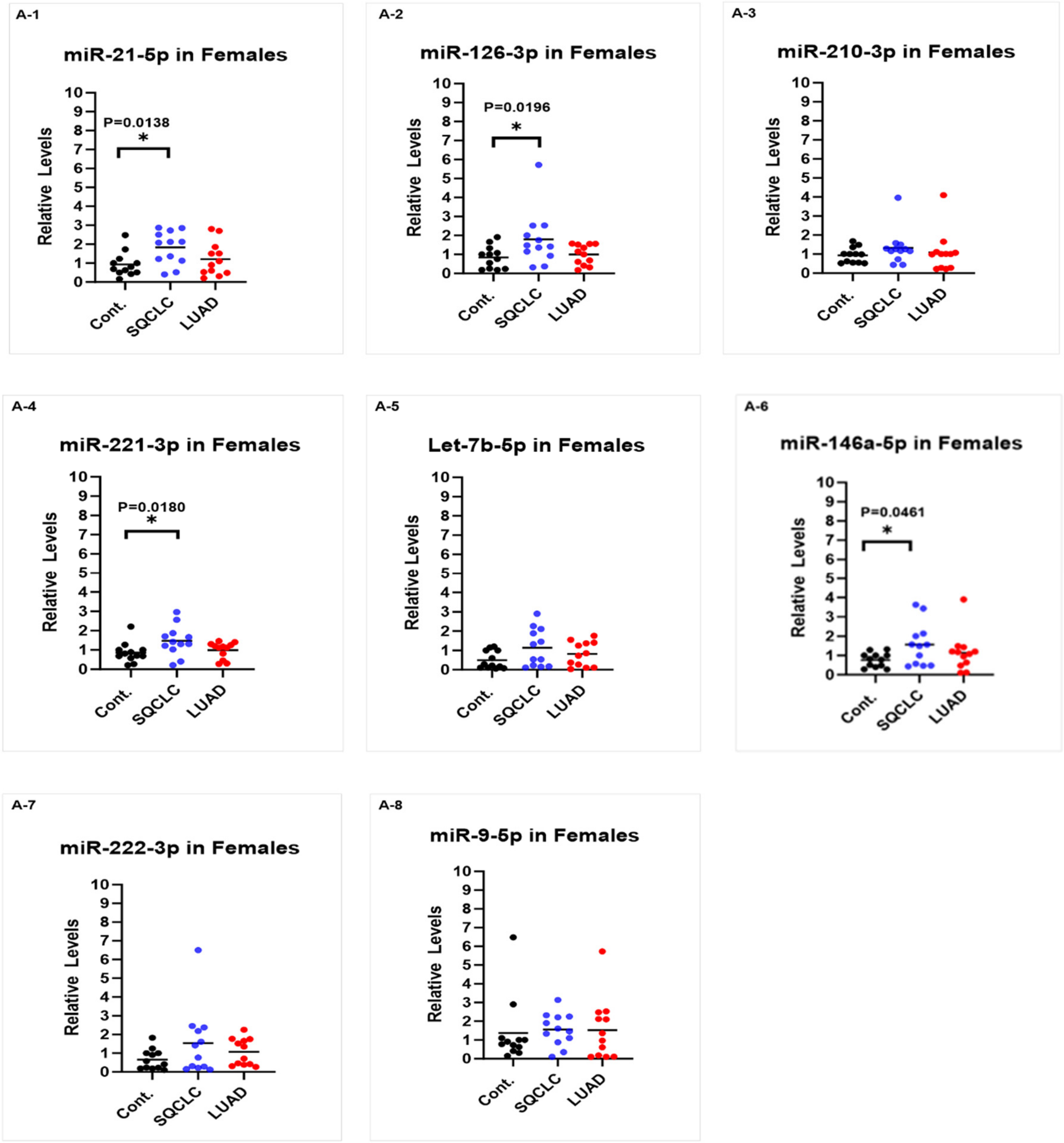

B

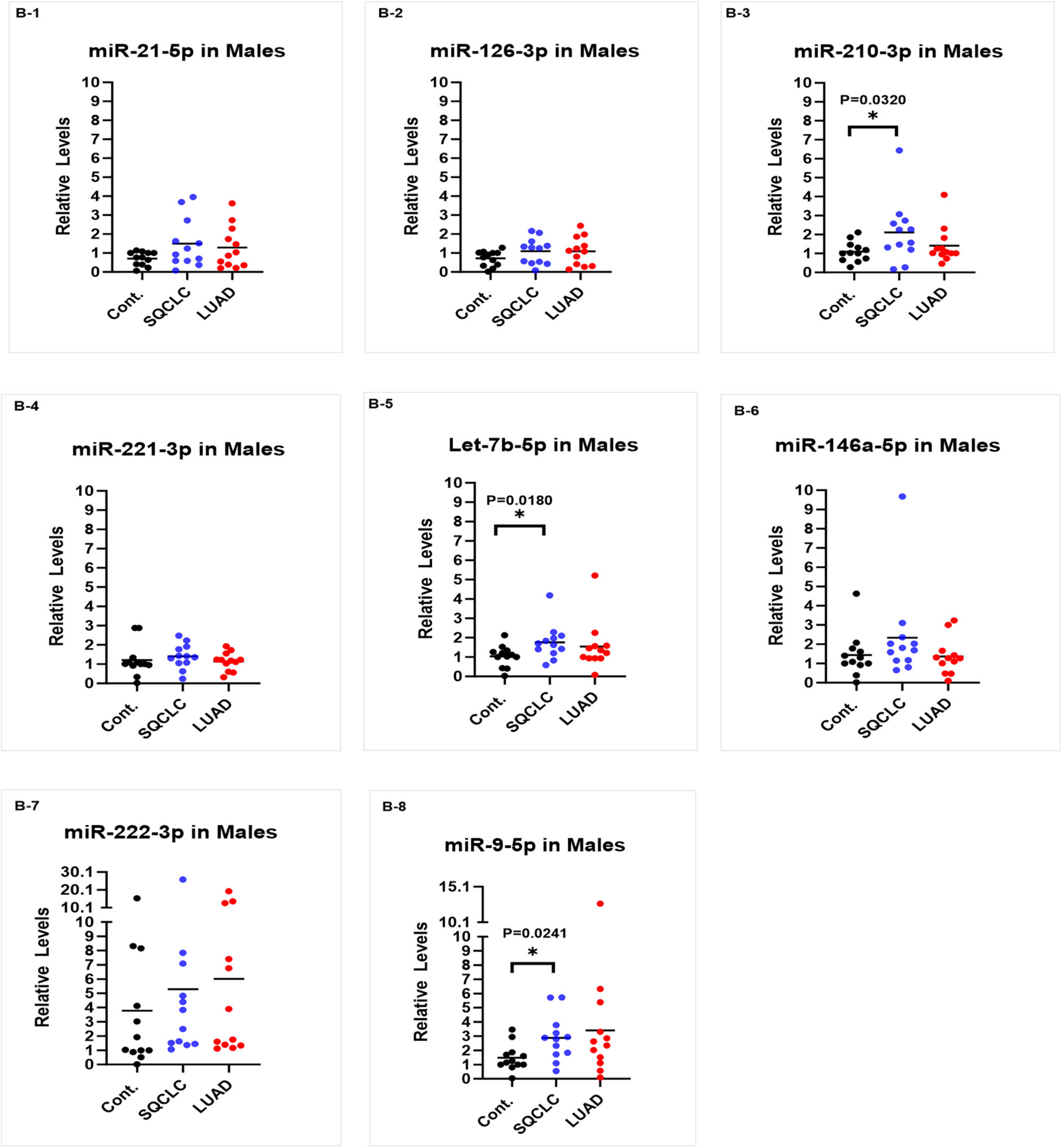
B-1
miR-21-5p in Males
Relative Levels
Cont.
SQCLC
LUAD
B-2
miR-126-3p in Males
Relative Levels
Cont.
SQCLC
LUAD
B-3
miR-210-3p in Males
P=0.0320
*
Relative Levels
Cont.
SQCLC
LUAD
B-4
miR-221-3p in Males
Relative Levels
Cont.
SQCLC
LUAD
B-5
Let-7b-5p in Males
P=0.0180
*
Relative Levels
Cont.
SQCLC
LUAD
B-6
miR-146a-5p in Males
Relative Levels
Cont.
SQCLC
LUAD
B-7
miR-222-3p in Males
30.1
20.1
10.1
Relative Levels
Cont.
SQCLC
LUAD
B-8
miR-9-5p in Males
15.1
10.1
P=0.0241
*
Relative Levels
Cont.
SQCLC
LUAD

C

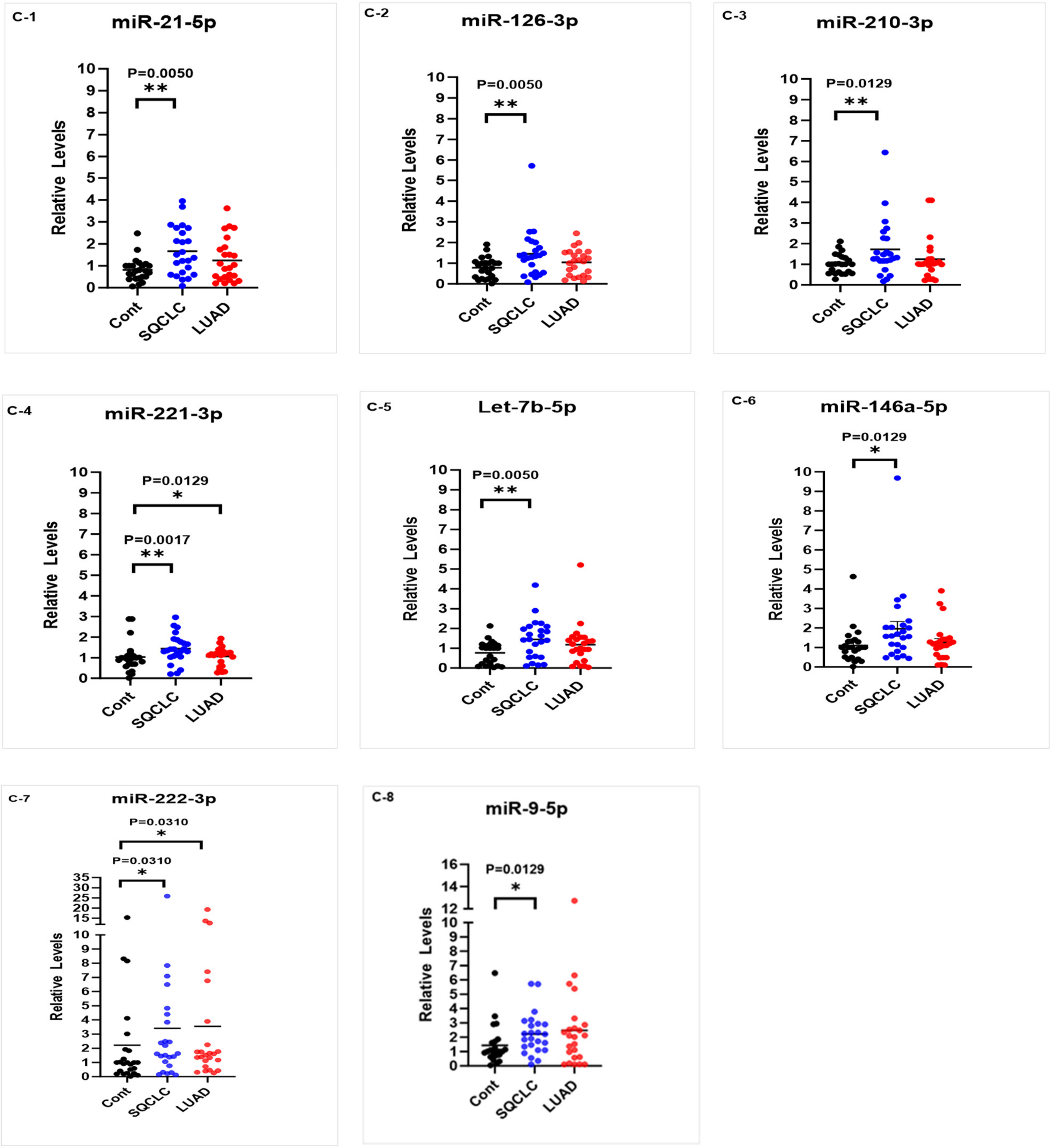
C-1
miR-21-5p
P=0.0050
**
Relative Levels
Cont
SQCLC
LUAD
C-2
miR-126-3p
P=0.0050
**
Relative Levels
Cont
SQCLC
LUAD
C-3
miR-210-3p
P=0.0129
**
Relative Levels
Cont
SQCLC
LUAD
C-4
miR-221-3p
P=0.0129
*
P=0.0017
**
Relative Levels
Cont
SQCLC
LUAD
C-5
Let-7b-5p
P=0.0050
**
Relative Levels
Cont
SQCLC
LUAD
C-6
miR-146a-5p
P=0.0129
*
Relative Levels
Cont
SQCLC
LUAD
C-7
miR-222-3p
P=0.0310
*
P=0.0310
*
Relative Levels
Cont
SQCLC
LUAD
C-8
miR-9-5p
P=0.0129
*
Relative Levels
Cont
SQCLC
LUAD

Supplementary Figure S2.

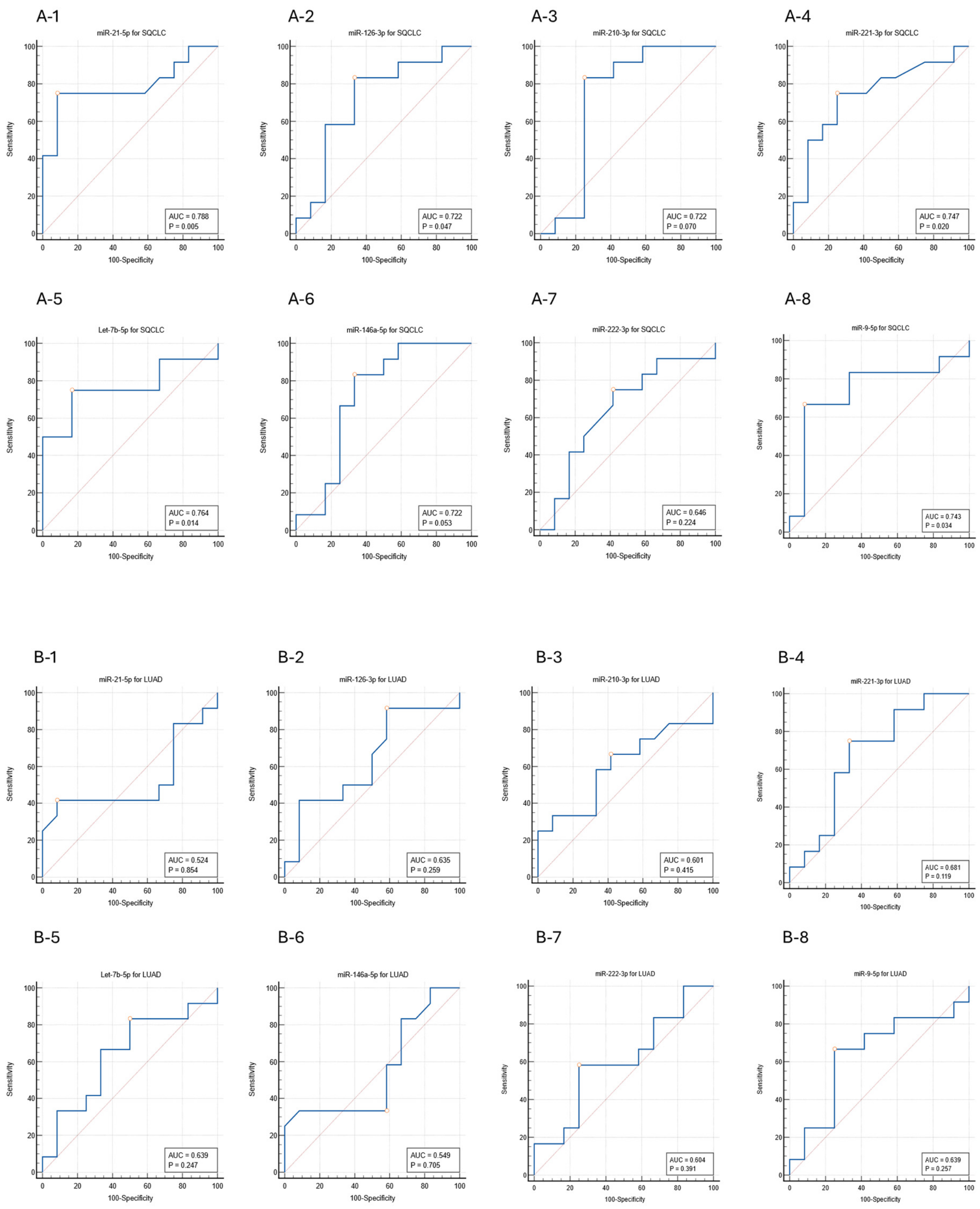

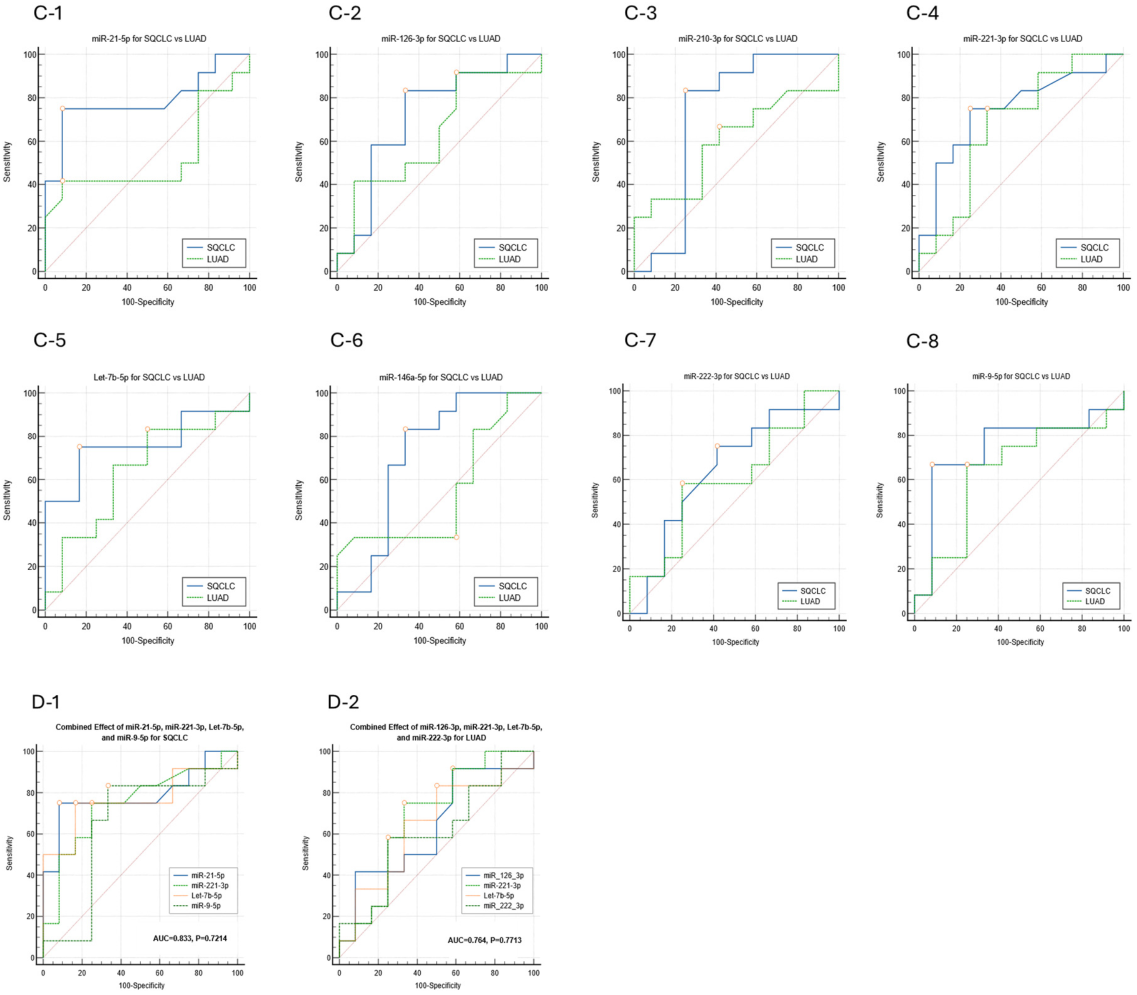

C-1
miR-21-5p for SQCLC vs LUAD
C-2
miR-126-3p for SQCLC vs LUAD
C-3
miR-210-3p for SQCLC vs LUAD
C-4
miR-221-3p for SQCLC vs LUAD
C-5
Let-7b-5p for SQCLC vs LUAD
C-6
miR-146a-5p for SQCLC vs LUAD
C-7
miR-222-3p for SQCLC vs LUAD
C-8
miR-9-5p for SQCLC vs LUAD
Sensitivity
100-Specificity
SQCLC
LUAD
D-1
Combined Effect of miR-21-5p, miR-221-3p, Let-7b-5p, and miR-9-5p for SQCLC
miR-21-5p
miR-221-3p
Let-7b-5p
miR-9-5p
AUC=0.833, P=0.7214
D-2
Combined Effect of miR-126-3p, miR-221-3p, Let-7b-5p, and miR-222-3p for LUAD
miR_126_3p
miR-221-3p
Let-7b-5p
miR_222_3p
AUC=0.764, P=0.7713
